# Findings of Gynecomastia That Developed in Follow-up Secondary to Bicalutamide Treatment on Bone Scan

**DOI:** 10.4274/mirt.galenos.2019.50490

**Published:** 2020-04-29

**Authors:** Kemal Ünal, Nahide Gökçora

**Affiliations:** 1 Acıbadem University, Department of Nuclear Medicine, İstanbul, Turkey; 2 Gazi University, Department of Nuclear Medicine, Ankara, Turkey

**Keywords:** Bicalutamide, gynecomastia, bone scan, prostate cancer

## Abstract

Prostate cancer is a common neoplastic disease especially in elder patients. Metastatic prostate disease has low five-year survival rate. Bicalutamide is an androgen receptor antagonist that acts as an inhibitor by competizing androgen receptors in the target tissue and used as a treatment option in prostate cancer. Bone scan was performed on a 79-year-old male with prostate cancer in our department. Blood pool images showed bilateral hyperemia in the breast regions which was not present on the previous scan one year ago. On physical examination, there was bilateral painful gynecomastia. It was learned that the patient was given Bicalutamide therapy after the first bone scan. Blood pool images may detect this side effect and should be evaluated with physical examination in case of clinical doubt.

## Abstract (tr)

Prostat kanseri, ileri yaşlarda görülme sıklığı artan ve uzak metastazı olan hastalarda 5 yıllık sağkalım oranı önemli ölçüde düşük seyreden bir neoplazidir. Bicalutamide, prostat kanserli hastalarda kullanılan ve hedef dokudaki androjen reseptörleri ile kompetisyona girerek inhibitör etki gösteren bir ilaçtır. Prostat kanseri nedeniyle takip edilen 79 yaşındaki erkek hastaya bölümümüzde kemik sintigrafisi çekildi. Kan havuzu görüntülerinde bir yıl önceki incelemesinde gözlenmeyen, her iki meme bölgesinde hiperemi gelişimi görüldü. Hastanın fizik muayenesinde bilateral ağrılı jinekomasti tespit edildi. İlk sintigrafisinden sonra hastaya Bicalutamide tedavisi başlandığı öğrenildi. Kan havuzu görüntüleri bu yan etkiyi tespit edebilir ve klinik şüphe halinde fizik muayene ile birlikte değerlendirilmelidir.

## Figures and Tables

**Figure 1:**
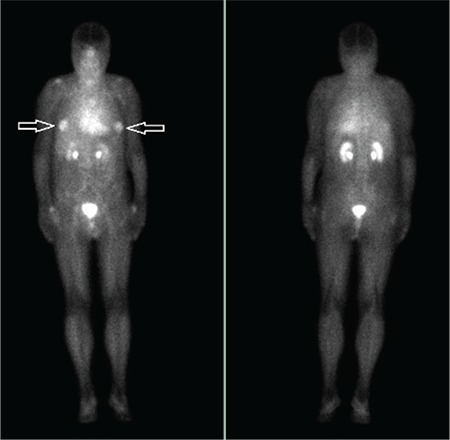
A 79-year-old male patient with prostate cancer underwent bone scan for the detection of bone metastases in our department. Blood pool images were taken immediately after the administration of Tc-99m labeled methylene diphosphonate and bone phase imaging was performed after 3 hours following the injection of radiopharmaceutical. In the blood pool phase, intense hyperemia was observed in regions corresponding to both areolar areas.

**Figure 2:**
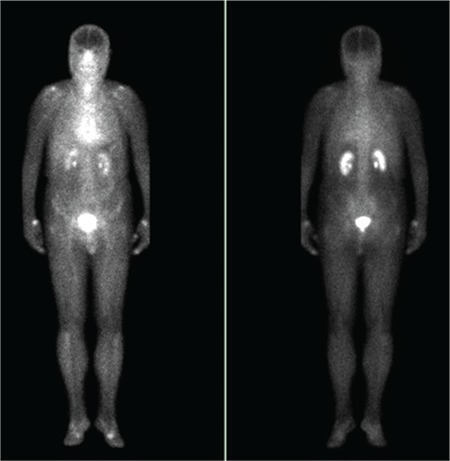
Previous scintigraphic examination of the patient one year before this scan and Bicalutamide treatment showed no sign of hyperemia in these areas.

**Figure 3:**
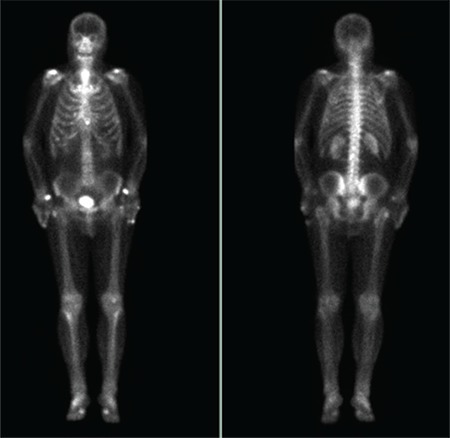
Bone phase images were consistent with stable degenerative changes and bone metastasis was not observed. Physical examination of the patient revealed bilateral painful gynecomastia. In the drug questionnaire, it was learned that the patient was given Bicalutamide therapy after the first bone scan. No other drug use or any disease that might cause gynecomastia was found. Bicalutamide is a nonsteroidal drug used in patients with prostate cancer, which can be administered in combination with LHRH analogues. It has a competitive inhibitor effect on cytosolic androgen receptors in the target tissue (1,2). Gynecomastia, breast pain, fatigue and decreased libido are common side effects of Bicalutamide (3,4). When an increase in estrogen/androgen ratio in the breast tissue occurs, this may cause gynecomastia and breast pain (5). When inflammation or swelling occurs in breast regions, this may be seen on blood pool images. If these hyperemic tissues or lesions have no effect on nearby bones, clearance of the radiopharmaceutical happens and normal distribution of the tracer is seen on late bone phase images. We routinely perform 2-phase imaging in oncology patients to analyze the possible hyperemic status of bone lesions for the differential diagnosis of benign or malignant pathologies. Physical examination may help narrowing the differential diagnosis among gynecomastia, other soft tissue lesions or radioactivity contamination, when this sign is seen. It is also important to keep in mind that gynecomastia can sometimes be seen unilaterally. There are also several reports in the literature about incidental gynecomastia and other nuclear medicine procedures apart from bone scan, such as sodium fluoride positron emission tomography (PET), myocardial single photon emission computed tomography or single photon emission computerized tomography PET imaging (6,7,8). Blood pool images may detect this side effect and should be evaluated with physical examination in case of clinical doubt.
